# 

**Published:** 1892-10

**Authors:** 


### CONTENTS.

ORIGINAL ARTICLES—Intestitial Nephritis—By W. O. Bridges, M. D., Omaha, Neb....523
Sexual Hypochondriasis—By C. Evans Johnson, M. D., Atlanta, Ga...533
The Radical Cure of Hernia, Particularly with Children—By Thos. H. Manley, A. M., M. D., New York.... 541
CORRESPONDENCE—Our London Letter....547
SOCIETY NOTES—Clinical Society of Maryland....550
EDITORIALS—Epidemic Cholera....564
Higher Medical Education....567
Southern Surgical and Gynecological Association—By Dr. Thos. F. Wood—To Our Readers....569
BOOK REVIEWS....553
Annual of the Univeısal Medical Sciences—Book on the Physician Himself—The Science and Art of Midwifery—Diseases of the Nervous System.
SELECTIONS AND ABSTRACTS—Incompatibles of Antipyrin....539
Lactic Acid as a Prophylactic in Gout....539
Dermatol in Gynecology....549
Treatment of Corneal Spots....552
Communication on the Treatment of Chronic Gonorrhœa....555
Change of Air and Baths in the Summer Diarrhœa of Children....556
Inoculation Against Cholera....556
The P.n Sensation in the Throat....557
Duties of Midwives....558
Treatmer.t of Uterine Hemorrhage....558
Injection of Testicle Juice in Tuberculosis....559
Headache during Childhood....559
Over Pressure in Children Causing Brain Mischief....561
PRESCRIPTION DEPARTMENT....574-575
Rheumatic Bronchitis—Pruritus Ani Vulvæ—Hepatic Eruption—Typhoid Fever—A Salve for Hemorrhoids—Acute Gout—Acute Gastritis—Antonic Dyspepsia—Vomiting of Pregnancy—Tonic after a Debauch.
SPECIAL NOTES....571-573

